# Comprehensive machine learning-based preoperative blood features predict the prognosis for ovarian cancer

**DOI:** 10.1186/s12885-024-11989-1

**Published:** 2024-02-26

**Authors:** Meixuan Wu, Sijia Gu, Jiani Yang, Yaqian Zhao, Jindan Sheng, Shanshan Cheng, Shilin Xu, Yongsong Wu, Mingjun Ma, Xiaomei Luo, Hao Zhang, Yu Wang, Aimin Zhao

**Affiliations:** 1 Department of Obstetrics and Gynecology, Renji Hospital, School of Medicine, Shanghai Jiaotong University, Shanghai, China; 2 Department of Obstetrics and Gynecology, Shanghai First Maternity and Infant Hospital, School of Medicine, Tongji University, Shanghai, China; 3 Shanghai Key Laboratory of Maternal Fetal Medicine, Shanghai First Maternity and Infant Hospital, School of Medicine, Tongji University, 200092 Shanghai, China

**Keywords:** Ovarian cancer, Machine learning, Blood features, Prognosis

## Abstract

**Purpose:**

Significant advancements in improving ovarian cancer (OC) outcomes have been limited over the past decade. To predict prognosis and improve outcomes of OC, we plan to develop and validate a robust prognosis signature based on blood features.

**Methods:**

We screened age and 33 blood features from 331 OC patients. Using ten machine learning algorithms, 88 combinations were generated, from which one was selected to construct a blood risk score (BRS) according to the highest C-index in the test dataset.

**Results:**

Stepcox (both) and Enet (alpha = 0.7) performed the best in the test dataset with a C-index of 0.711. Meanwhile, the low RBS group possessed observably prolonged survival in this model. Compared to traditional prognostic-related features such as age, stage, grade, and CA125, our combined model had the highest AUC values at 3, 5, and 7 years. According to the results of the model, BRS can provide accurate predictions of OC prognosis. BRS was also capable of identifying various prognostic stratifications in different stages and grades. Importantly, developing the nomogram may improve performance by combining BRS and stage.

**Conclusion:**

This study provides a valuable combined machine-learning model that can be used for predicting the individualized prognosis of OC patients.

**Supplementary Information:**

The online version contains supplementary material available at 10.1186/s12885-024-11989-1.

## Introduction

Ovarian cancer(OC), an aggressive gynecological cancer, has a 5-year survival rate of less than 50% and ranks the first in tumor-related deaths among gynecologic cancers in the United States [1]. 75% of epithelial ovarian cancer (EOC) patients are already in advanced stages at the time of detection due to the sneaky clinical symptoms and the lack of early screening tools [2]. Traditional FIGO stage, grade, CA-125, and tumor residuals provide a relatively reliable reference for patient treatment selection and predicting prognosis [3–7]. Still, the high degree of heterogeneity in EOC, even among patients at the same stage, can lead to a wide range of outcomes [8].

In recent years, treatment for ovarian cancer is no longer a ‘one-size-fits-all’ fixed treatment proposition [9, 10]. Multiple clinical trials have categorized patients into high- and low-risk cohorts [11]. In many cases, the treatment decision with PARP inhibitors or bevacizumab may be influenced by the risk stratification predicted based on these clinical and genetic factors [11]. Therefore, the construction of predictive models for EOC prognosis is essential.

Through bioinformatics research, numerous prognostic models for gene signatures have been developed. These prognostic models, while generally achieving good predictive results, lacked integrated biological signatures because they were based on gene expression files for specific biological pathways such as immune [12], metabolism [13], m6A [14], and autophagy [15]. As a result, there is a need to take into account more usable and effective clinical biomarkers for prediction. Previous research successfully predicted prognosis using various preoperative blood indicators [16, 17]. According to certain research, blood indicators may represent the tumor microenvironment. However, screening using a few blood indicators resulted in the loss of key information and was inadequate for exploring the characteristic landscapes and survival prognosis of OC patients. Machine learning (ML) has shown enormous application value in evaluating prognosis and making clinical diagnoses. Besides, ML can adequately utilize large datasets for training, which avoids loss of data. Previous studies have demonstrated the superiority of machine learning algorithms over non-machine learning algorithms [18–25]. Using a decision tree algorithm, Feng et al. [25] constructed a prediction model for EOC based on preoperative blood markers and clinicopathologic parameters, but the prediction still has greater potential for improvement. Using multiple types of machine learning algorithms, the integrated program could provide a model with consensus output for OC prognosis. And the combination of algorithms can further reduce the dimensionality of the variables, making the model more simplified and increasing accuracy. Previously, Hansen et al. identified and quantified circRNAs expression by combining two (or more) algorithms and found that algorithm combinations could improve algorithm complementarity and resolve algorithm-specific false positives [26].

Here, we utilized 88 machine learning algorithm combinations to explore prognostic stratification based on blood features to guide individualized management of EOC patients.

## Methods

### Study population

**Fig. 1:**
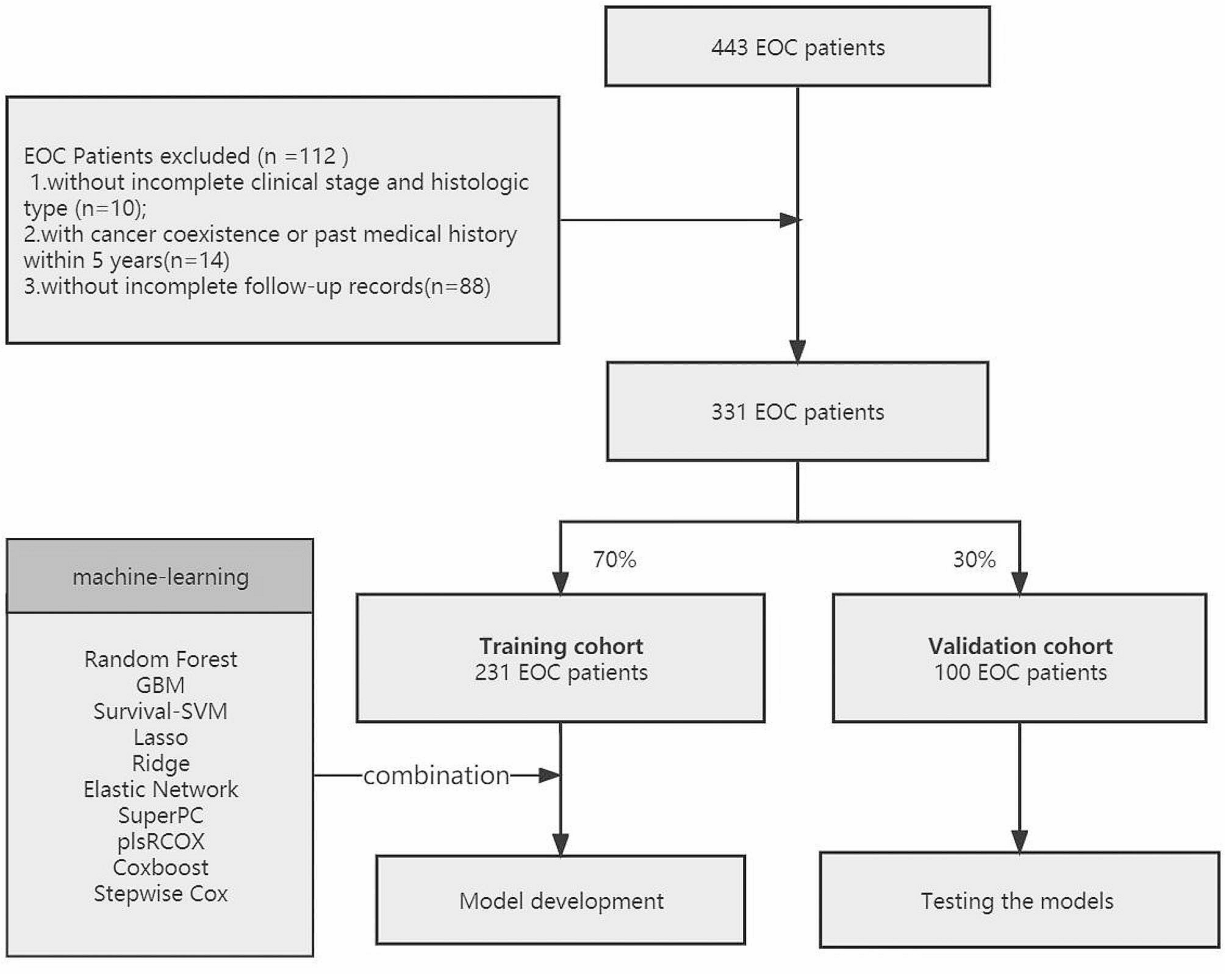
Study design process

Figure 1 depicted the research design process schematically. Retrospective screening was performed on 443 EOC patients from Jan.2010 to Dec.2020. Exclusion criteria were as described in previous articles [27]. In addition to this, patients with no follow-up records were excluded (*n* = 88). Finally, a total of 331 EOC patients were matched. The original dataset (*n* = 331) was randomly divided into training dataset (*n* = 231) and test dataset (*n* = 100) using a 7:3 ratio. The analysis has been approved by the Ethics Committee of Renji Hospital Affiliated to Shanghai Jiao Tong University School of Medicine.

### HRD status assessments

Combining the homologous recombination deficiency (HRD) score and the status of BRCA1/2 mutations is necessary to determine the HRD status. The HRD score was calculated as the sum of the loss of heterozygosity (LOH) , telomeric allelic imbalance (TAI), and large-scale state transitions (LST) scores. HRD score was tested by BGI Genomics Co., Ltd., and HRD status was considered positive if the HRD score was greater than 42 or BRCA1/2 mutations.

### Prognostic signature selection and development

Based on our previous study [27], a total of 33 blood features were selected from the cohort. Next, to develop the blood risk score (BRS), we incorporated these blood features and age into our program. The preoperative blood features contained Sodium (Na), Potassium (K), Chlorine (Cl), White blood cell (WBC), Neutrophil (Neu), Lymphocyte (Lym), Hematocrit (Hct), and Platelet (PLT), Red blood cell (RBC), Hemoglobin (Hb), Monocyte (Mono), Eosinophil (Eo), Basophil (Baso), Carcinoembryonic antigen (CEA), Alpha-fetoprotein (AFP), Carbohydrate antigen 19 − 9 (CA19-9), and Carbohydrate antigen 125 (CA-125), Blood urea nitrogen (BUN), Creatinine (Cr), Uric acid (UA), Alanine aminotransferase (ALT), Aspartate aminotransferase (AST), Alkaline phosphatase (ALP), Total protein (TP), Albumin (Alb), Prealbumin (PA), globulin (GLOB), glutamyl transpeptidase (GGT), Lactate dehydrogenase (LDH), Thrombin time (TT), Prothrombin time (PT), Fibrinogen (Fb) and Activated partial thromboplastin time (APTT).

To enhance the accuracy and robustness of comprehensive and systematic approaches, we integrated 10 ML algorithms and generated 88 algorithm combinations. We used the preceding procedure to create a reliable prognosis model for ovarian cancer [28, 29]. In this study, we combined 10 classical algorithms: random forest (RSF), survival support vector machine (Survival-SVM), least absolute shrinkage and selection operator (LASSO), elastic network (Enet), gradient boosting machine (GBM), supervised principal components (SuperPC), ridge regression, partial least squares regression for Cox (plsRcox), CoxBoost, and Stepwise Cox (StepCox). Variable screening was provided by RSF, LASSO, CoxBoost, and Stepwise Cox. We constructed 88 model combinations using the framework of LOOCV based on the approach of Liu et al. [30]. Next, we constructed prognostic signatures in the training dataset using a combination of 88 models. To train and tune the models, and reduce overfitting, the original training dataset was divided into a sub-training set and a validation set through LOOCV. Specifically, in each LOOCV trial, N-1 samples were used as the sub-training set to train the models, and the remaining single sample was used as a validation set to validate the models and optimize model parameters. This process was repeated N times until each sample was used as a validation set once. When the models were obtained, we evaluated the models using the test dataset. We used prognostic models to predict the overall survival of patients. The BRS was finally estimated using the signatures gathered from the training and test cohorts. More details were shown in the Supplementary Material.

### Evaluating the clinical significance of BRS

The concordance index (C-index) and the integrated Brier score (IBS), two widely used assessment metrics, were employed in the prior papers to assess the efficacy of the survival prediction model [31]. C-index is defined as the proportion of patient pairs in which the predicted and observed survival outcomes were concordant [32]. A C-index of 0.5 indicates no predictive discrimination, and a C-index of 1 indicates perfect predictive accuracy. The IBS, which represents the mean squared discrepancies between observed survival status and anticipated survival probability at a specific time point, is used to assess the error of survival prediction. An IBS value of 0 suggests perfect prediction, whereas 1 shows completely wrong prediction. By taking into account the highest C-index of the test cohort, we were able to determine the best prognostic model for OC. In addition, the Mean Square Error (MSE) of the training dataset was calculated based on the predicted results generated by each iteration of LOOCV. The MSE of the test dataset was calculated based on the final model. The smaller the MSE value, the more accurate the predicted results.

Between high- and low-risk groups, clinical parameters such as age, FIGO stage, and grade were compared. A Kaplan-Meier (KM) analysis in clinical subgroups was also conducted. To evaluate the BRS’s predictive power, receiver-operator characteristic (ROC) curves were created for the test dataset. We conducted time-dependent ROC curves and areas under the curve (AUCs) analyses of the model predictive power at 3, 5 and 7 years using the R package timeROC. We used SHAP to interpret the output of the optimal machine learning combination [33].

### Construction of nomogram

Multivariate and univariate analyses were carried out using Cox’s hazards regression model. Hazard ratios (HR) were determined from Cox proportional hazards regression models. And the prognostic risk factor is indicated by an HR more than 1, whereas the protective impact is shown by an HR less than 1. The “rms” package of the R software was used to create the nomogram. To assess the discrimination of the nomogram model, time-ROC and calibration curves were used.

### Statistical analysis

The R software (v.4.1.3) was used for all statistical analysis. Categorical variables were analyzed using the chi-squared or Fisher exact tests, while continuous variables were studied using the Wilcoxon rank-sum or T tests. The ROC analysis was performed using the R package “survivalROC”, and the optimal cut-off value of BRS for predicting overall survival (OS) was determined. There was statistical significance at *P* < 0.05.

## Results

### Clinical characteristics

**Fig. 2:**
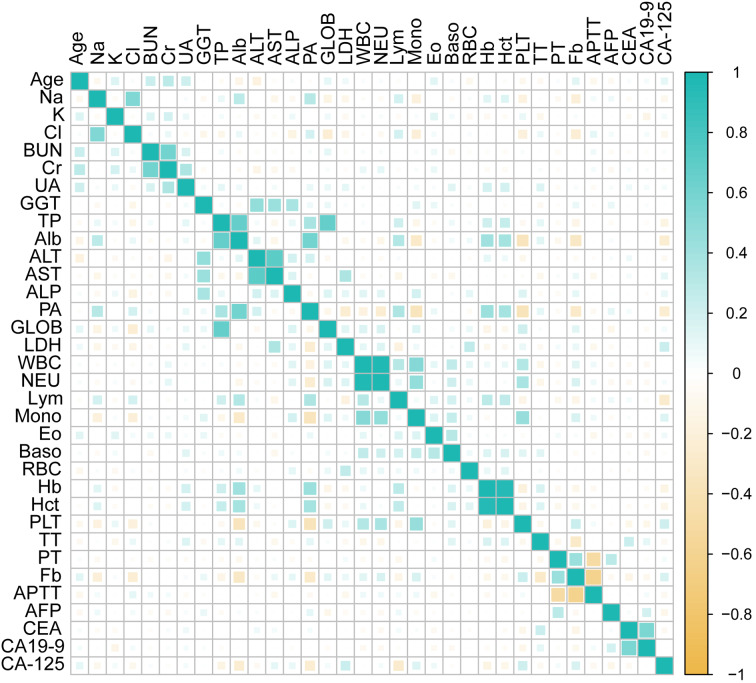
The correlation heat map. The correlation between the biomarkers was depicted in the heatmap

Table 1 listed the general clinical characteristics of the EOC patients. The mean age of datasets was 57.61 ± 10.39 years old. A total of 137 (41.4%) and 194 (58.6%) patients were in early (FIGO I or II) or late (FIGO III or IV) stages of the OC. Histology-proven serous subtypes were present in 229 (70.2%) of patients. A heat map was obtained to express the results of Pearson correlation analysis of selected features (Fig. 2).

**Table 1:** The baseline characteristics of the EOC patients

	Overall(*n* = 331)	Training(*n* = 231)	Test(*n* = 100)	P value
Age (mean ± SD)	57.61 ± 10.39	57.89 ± 10.06	56.98 ± 11.14	0.466
**Stage (%)**				0.214
Early	137 (41.4)	90 (39.0)	47 (47.0)	
Late	194 (58.6)	141 (61.0)	53 (53.0)	
**Histologic types (%)**				0.058
Serous	229 (69.2)	158 (68.4)	71 (71.0)	
Endometrioid	48 (14.5)	36 (15.6)	12 (12.0)	
Mucinous	43 (13.0)	33 (14.3)	10 (10.0)	
Clear cell	11 (3.3)	4 (1.7)	7 (7.0)	
**Grade (%)**				0.823
G1	13 (3.9)	8 (3.5)	5 (5.0)	
G2	44 (13.3)	31 (13.4)	13 (13.0)	
G3	261 (78.9)	184 (79.7)	77 (77.0)	
NA	13 (3.9)	8 (3.5)	5 (5.0)	

### Univariate and multivariate Cox analysis

We conducted univariate and multivariate Cox regression analysis in all patients to further ascertain whether these chosen features acted as an independent risk factor for the survival outcome of ovarian cancer patients (Table 2). The UA (HR = 1.0031, *P* = 0.0279), TP (HR = 0.9568, *P* = 0.0018), Alb (HR = 0.9159, *P* = 0.0000), AST (HR = 1.0212, *P* = 0.0146), PA (HR = 0.9955, *P* = 0.0062), LDH (HR = 1.0015, *P* = 0.0112), Lym (HR = 0.6378, *P* = 0.0435), Hct (HR = 0.0036, *P* = 0.0344), TT (HR = 0.8264, *P* = 0.0001), Fb (HR = 1.0908, *P* = 0.0347), and CA-125 (HR = 1.0002, *P* = 0.0006) were determined as significantly prognostic factors for OS through the univariate analysis (Table 2). We used multivariate cox regression analysis to adjust for any potential confounding factors that may have existed in univariate cox’s regression. Finally, UA (HR = 1.0044, *P* = 0.013), Alb (HR = 0.7742, *P* = 0.0117), TT (HR = 0.7805, *P* = 0.0000), and CA-125 (HR = 1.0002, *P* = 0.01) were independent factors for survival according to multivariate cox regression analysis.

**Table 2:** Univariate and multivariate cox regression analysis

Characteristic	Univariate analysis				Multivariate analysis			
	HR	CI5	CI95	P	HR	CI5	CI95	P
Age	1.0185	0.9961	1.0414	0.1067	1.003	0.976	1.0307	0.8312
Na	0.9589	0.904	1.0172	0.1633	0.9752	0.8964	1.061	0.5591
K	0.8446	0.456	1.5642	0.5912	0.7335	0.3617	1.4877	0.3903
Cl	0.9739	0.9221	1.0286	0.3429	0.997	0.923	1.0769	0.9386
BUN	1.0067	0.9379	1.0805	0.8543	0.9542	0.8397	1.0842	0.4718
Cr	1.005	0.9933	1.0169	0.403	1.0103	0.9896	1.0314	0.3323
UA	1.0031	1.0003	1.0059	0.0279	1.0044	1.0009	1.0079	0.013
GGT	1.0051	0.9996	1.0107	0.0699	1.0141	1.0043	1.024	0.0048
TP	0.9568	0.9306	0.9838	0.0018	1.1998	0.9893	1.4551	0.0642
Alb	0.9159	0.8818	0.9512	0	0.7742	0.6346	0.9446	0.0117
ALT	1.0016	0.9875	1.0158	0.8278	0.9981	0.9737	1.0231	0.8799
AST	1.0212	1.0041	1.0386	0.0146	0.9825	0.9463	1.02	0.3545
ALP	0.9965	0.9885	1.0045	0.3889	0.9896	0.981	0.9983	0.0195
PA	0.9955	0.9923	0.9987	0.0062	0.9961	0.9912	1.001	0.1215
GLOB	0.9841	0.942	1.0281	0.4728	0.8155	0.6683	0.9951	0.0447
LDH	1.0015	1.0004	1.0027	0.0112	1.0009	0.9987	1.003	0.4265
WBC	0.987	0.9004	1.082	0.7806	0.1228	0.0034	4.4342	0.2518
NEU	1.0093	0.92	1.1073	0.8451	8.435	0.2317	307.0629	0.245
Lym	0.6378	0.4122	0.9869	0.0435	7.6662	0.2157	272.4187	0.2635
Mono	1.2825	0.374	4.3978	0.6923	7.557	0.1072	532.9701	0.3517
Eo	0.1132	0.0047	2.751	0.1808	0.3733	0.0032	43.7731	0.6852
Baso	0	0	1.5958	0.0573	0.0421	0	2413175.62	0.7282
RBC	0.7635	0.5509	1.0581	0.1051	1.0206	0.7806	1.3344	0.8814
Hb	0.9871	0.9732	1.0011	0.0716	1.0303	0.9668	1.0981	0.3576
Hct	0.0036	0	0.6605	0.0344	0	0	78971.0957	0.3161
PLT	1.0006	0.9984	1.0029	0.5785	0.9984	0.9953	1.0014	0.295
TT	0.8264	0.7499	0.9107	0.0001	0.7805	0.6947	0.8769	0
PT	1.0013	0.9765	1.0266	0.92	0.9648	0.8858	1.0509	0.411
Fb	1.0908	1.0062	1.1824	0.0347	0.9144	0.7729	1.0817	0.2964
APTT	0.984	0.9576	1.0112	0.2455	0.9459	0.892	1.0031	0.0633
AFP	0.9648	0.8779	1.0603	0.4568	1.0154	0.9012	1.144	0.802
CEA	1.0114	0.9888	1.0346	0.3255	1.0462	1.0074	1.0865	0.0193
CA19-9	0.9999	0.9995	1.0003	0.4992	0.9994	0.9988	1.0001	0.1087
CA-125	1.0002	1.0001	1.0003	0.0006	1.0002	1	1.0003	0.01

### Integrated development of ovarian cancer prognosis model

ML with preoperative blood metrics as input was trained to export a risk score for survival, which was used to measure the level of risk for an individual. For our training cohort, we implemented 88 algorithm combinations to acquire prediction models, then, for our test cohort, we calculated the C-index and IBS of each algorithm. Considering there were fewer independent predictors and the model had a filtering function, we did not use the above independent risk factors to train models but instead used all the characteristics.

**Fig. 3:**
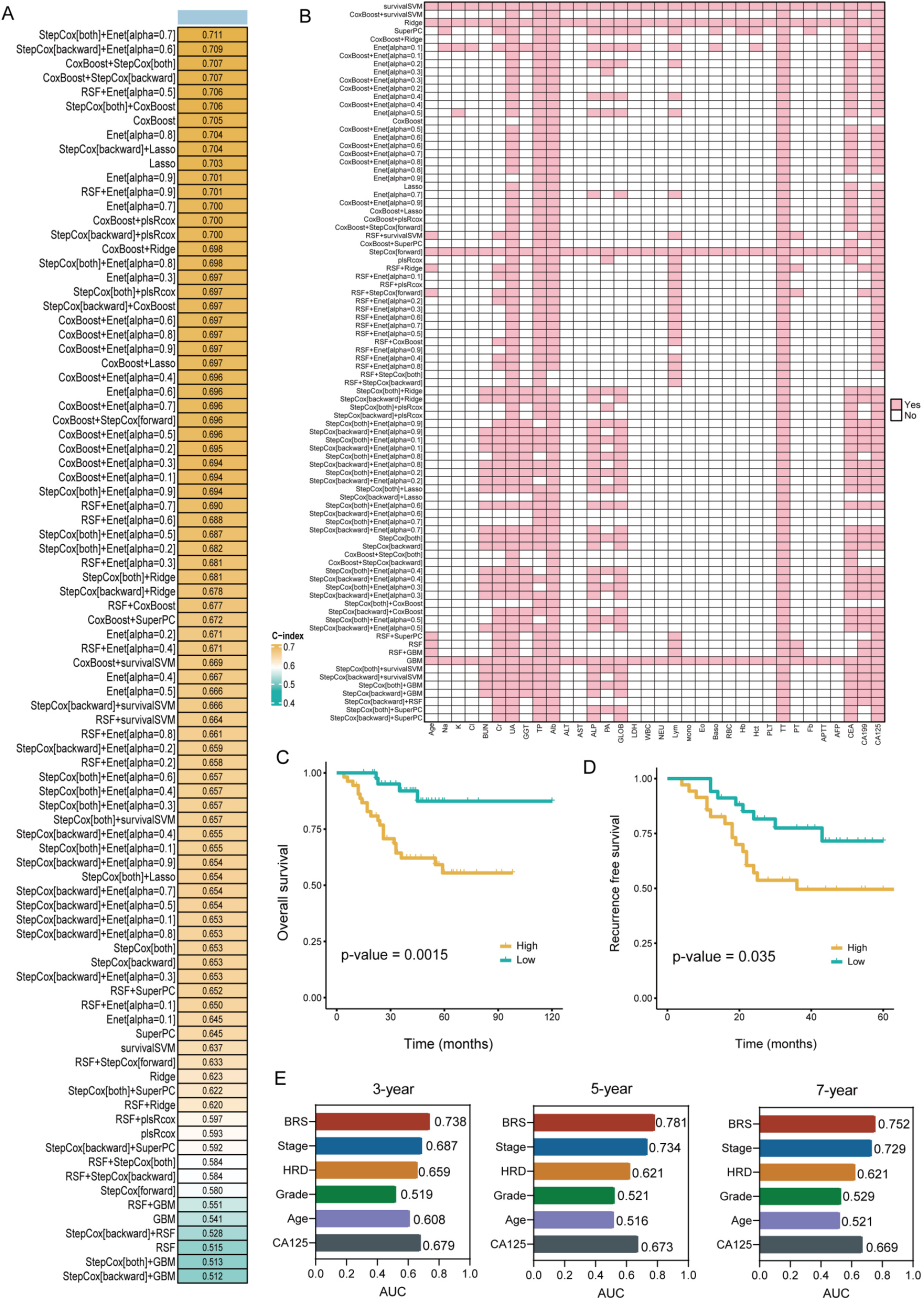
Construction and testing of the combination machine learning model-based blood features for prognosis of OC patients. **A**. The C-index values for 88 ML algorithms were calculated in the test dataset. **B.** Selection of blood features for developing machine-learning models. **C**, **D**. KM survival analysis for overall survival (**C**) and recurrence free survival (**D**) between the high and low BRS groups in the test dataset. **E**. Common clinical characteristics at 3,5,7 years in the test dataset were contrasted with the predict performance of BRS.

As shown in Fig. 3A and Table S1, the combination of Stepcox (both) and Enet (alpha = 0.7) with the most prominent C-index (0.711) and the low IBS (0.169) was chosen as the final model. The mean MSE in the training dataset was 0.188, and the test dataset was 0.192. Following final model evaluation, we calculated BRS for every sample in the test cohort. The characteristics used by each model were shown in Fig. 3B. The features selected for the optimal model were TP, Alb, and TT. In Figure S1, we presented the SHAP values of features for the optimal model. BRS was categorized based on its cut-off value (0.007) into high and low groups to evaluate its prognostic performance. A KM curve for OS and RFS shows that the high BRS group had significantly shorter survival times in the test cohort (*p* = 0.0015 for OS and *p* = 0.035 for RFS, Fig. 3C and D). To measure the discrimination of BRS, we conducted the analysis of time-ROC. In the test group, the 3-, 5-, and 7-year OS of BRS had respective AUCs of 0.738, 0.781, and 0.752. which was higher than other common prognostic predictors, such as FIGO stage, HRD status, grade, age, and CA-125 (Fig. 3E).

### Predictive performance in different clinical features

**Fig. 4:**
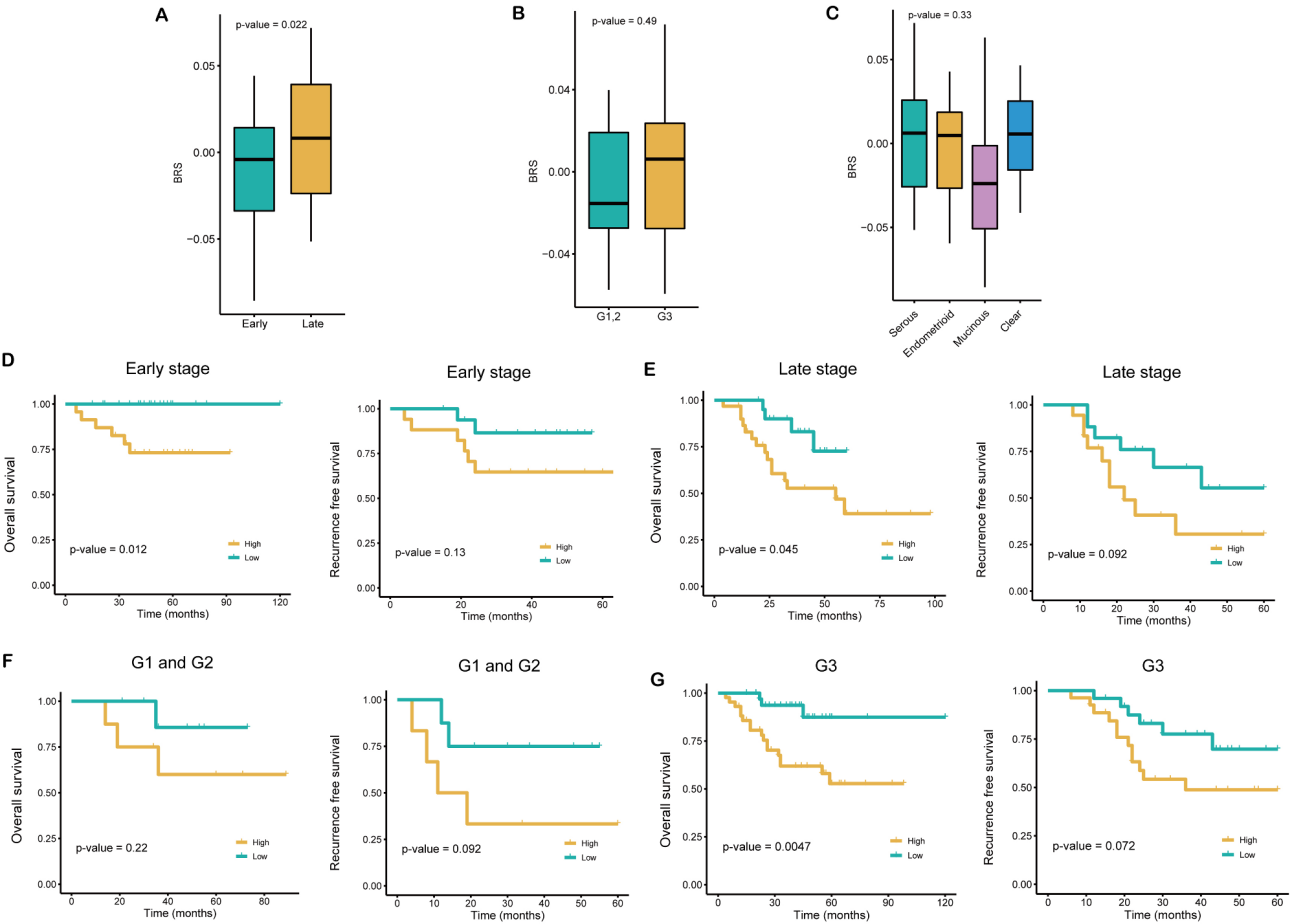
Performance of BRS in different subgroups. **A**, **B**, **C**. The distribution of BRS in stage (**A**), grade (**B**), and histologic types (**C**). **D**, **E**. The KM analysis of overall survival and recurrence free survival in different stage subgroups. **F**, **G**. The KM analysis of overall survival and recurrence free survival in different grade subgroups

In order to better understand BRS, we grouped patients of the test dataset based on several clinical traits, including stage, grade and pathology type. At the same time, subgroup analysis reduced the presence of heterogeneity and allowed for more reliable prediction results. With the later FIGO stage, we discovered that the BRS significantly increased (*p* = 0.022), but there were no significant differences in grade and pathology type (Fig. 4A-C). Interestingly, BRS also significantly improved the capacity to distinguish overall survival in several clinical subgroups, such as stage (early and late) and G3 group, although no differentiation was demonstrated for the RFS (Fig. 4D-G). In these subgroups, high BRS represented poorer overall survival.

### Nomogram based on BRS and clinical features

**Fig. 5:**
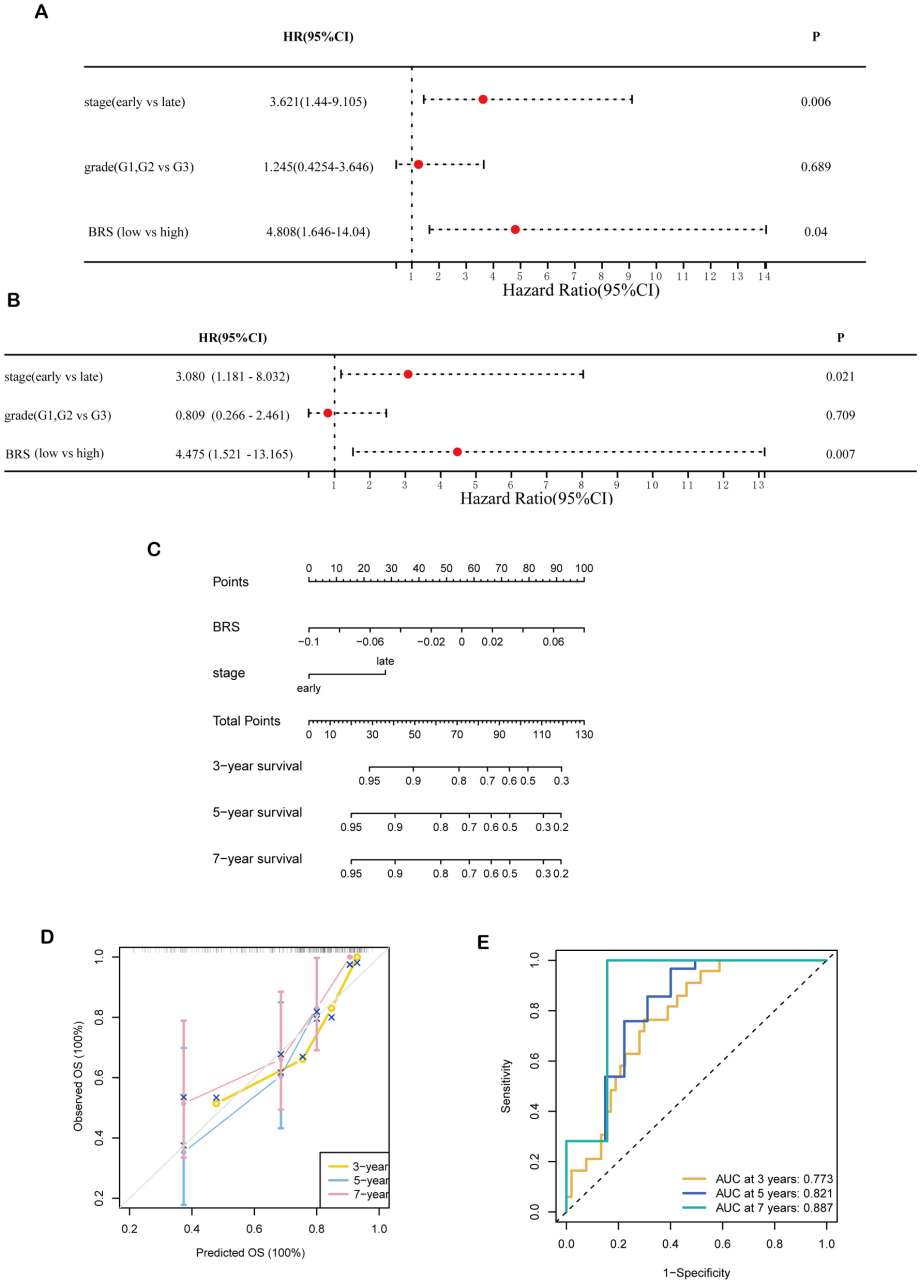
The development of nomogram. **A**, **B**. Univariate (**A**) and multivariate (**B**) cox regression analysis. **C**. The nomogram integrated BRS and stage was constructed. **D**. Calibration curves used to compare the predicted and actual 3, 5,7 years survival probabilities. **E**. Time-dependent receiver-operator characteristic (ROC) analysis for predicting 3-, 5-, and 7-year OS.

Using univariate Cox regression analysis, we identified BRS (HR = 4.808, *P* = 0.04) and stage (HR = 3.621, *P* = 0.006) as risk factors for OS (Fig. 5A). Furthermore, through multivariate cox regression, we found that BRS (HR = 4.475, *P* = 0.007) and stage (HR = 3.08, *P* = 0.021) were independent risk factors (Fig. 5B). Given the prospective therapeutic applicability of BRS, a predictive nomogram incorporating two independent predictors of mortality (BRS and stage) was constructed (Fig. 5C). Meanwhile, personalized patient scores were computed to predict the OS at three, five, and seven years. According to the calibration plot, our nomogram performed good in predicting OC patients’ prognoses (Fig. 5D). At 3-, 5-, 7-year, the nomogram’s AUCs were 0.773, 0.821, and 0.887 (Fig. 5E), which indicated its accuracy and stability. In addition, the nomogram model had an IBS of 0.153 (Table S2). whereas the BRS has an IBS of 0.169. Thus, the excellent predictive performance of nomogram model for long-term survival was validated.

## Discussion

Conventional features have considerable limitations in prognostic management and hazard rate estimation when taken into account in the context of tumor heterogeneity and the varied clinical outcomes of patients at the same stage. Since many patients were not analyzed genomically, many prognosis markers or predictive models could not be directly used for clinical application. To ascertain the prognosis of OC, it may be beneficial to investigate the classification and risk stratification of tumors by making adequate use of clinically available blood tests.

A growing amount of research has shown that peripheral blood test was essential for determining the prognosis of ovarian cancer [34–36] and other malignant cancers [37–39]. Preoperative blood markers can be quickly identified using standard blood testing, which is more convenient and affordable. However, preoperative peripheral blood assessment systems of ovarian cancer are not yet complete. Since 2000, there has been a tremendous improvement in the accuracy of employing ML models to predict patient survival and diagnosis [40]. Our previous article established supervised diagnostic models and unsupervised prognosis models based on age and pre-operative blood indicators. To further increase the predictive power of the model, we constructed prognostic characteristics of OC patients by combining machine learning algorithms using age and 33 blood metrics. In order to prevent unsuitable model approaches owing to personal preferences, we combined 10 machine learning algorithms into 88 combinations and chose the best model. This combined ML model approach has been used to predict the prognosis of bladder cancer [41], muscle-invasive urothelial cancer [42], pancreatic cancer [29], and endometrial cancer [43], as well as validated in multiple datasets with good robust and AUC values. Importantly, the optimal model demonstrated strong and stable prediction performance by evaluating the C-index, IBS, and mean MSE. Three, five-, and seven-year OS in the test cohort had AUCs of 0.738, 0.781, and 0.752, respectively. This predictive efficacy was superior to our risk model based on unsupervised machine learning [27]. Surprisingly, the predictive efficacy of BRS exceeded that of models integrating multi-scale clinical imaging and genomic data [44]. Some traditional clinical characteristics have been shown to be useful in the prognostic assessment of OC patients. Therefore, we contrasted the effectiveness of BRS with these clinical characteristics. Apparently, the predictive efficacy of our model was preferable to these traditional predictors, including age, pathological grade, stage, HRD status, and CA-125. We compared BRS with currently recognized prognostic biomarkers in clinical practice and guidelines, which also increased the trust of physicians in our model.

The features identified by our optimal model included TP, Alb, TT. Zhong et al. found that thrombin could induce epithelial-mesenchymal transition and promote the invasion of ovarian cancer cells [45]. A recent study has revealed the connections between OC growth and coagulation [46]. Our study emphasized the importance of TT for the prognosis of OC, which may provide new insights into the biological mechanisms of coagulation in ovarian cancer. Serum Alb level is a crucial indicator for patients’ systemic inflammatory response and nutritional condition. The relationship between Alb level and the prognosis of patients has been found in many cancers, including ovarian, colorectal, and lung cancer [47]. The effect of albumin on ovarian cancer is complex, and additional approaches are needed to explore the mechanisms.

Importantly, the stage between the high- and low-risk groups varied significantly. We discovered that as FIGO stage was raised, risk scores considerably rose. Besides, BRS significantly improved the capacity to identify different clinical subgroups’ survival statuses. Our model exhibited independent predictive performance after adjusting for stage and grade. The nomogram was further modified to increase the clinical utility of BRS. It showed higher AUC values compared to BRS alone, and exceeding the predictive power of a nomogram also based on peripheral blood features constructed by Bai et al. [48], implying a higher predictive value for prognostic prediction in OC patients, which suggested that it may be a promising alternative metric for assessing prognostic risk in clinical OC.

However, BRS still has some limitations. First, all of the samples used in our investigation were retrospective, thus prospective samples should be used in the future for BRS corroboration. Second, we accept that our work will need external validation because it was only evaluated on a dataset from one institution. Finally, the lack of investigation into therapy efficacy needs more confirmation in the future, and exploring integrated genomes and imaging models with BRS could improve risk stratification’s ability to predict outcomes.

In summary, we combined various ML methods to predict risk stratification for EOC patients, and we found that the integrated algorithms increased the efficacy of the test dataset beyond common clinical factors. Our findings promoted clinical prognostic research by multiple combination machine learning.

### Electronic supplementary material

Below is the link to the electronic supplementary material.

Supplementary Material 1

Supplementary Material 2

Supplementary Material 3

Supplementary Material 4

## Data Availability

These findings’ data are now being used in another study, thus, they cannot be shared. Following the publication of the paper, requests for data will be taken into consideration by the corresponding author.

## Code Availability

The corresponding author can provide the code used to produce the findings in this study upon request.
