# Supplementary material for: Comprehensive machine learning-based preoperative blood features predict the prognosis for ovarian cancer
Source: BMC Cancer. 2024 Feb 26;24:267. doi: 10.1186/s12885-024-11989-1 (PMC10895771; doi:10.1186/s12885-024-11989-1)
Supplement: Supplementary file 1 — Supplementary Material 1 [file 12885_2024_11989_MOESM1_ESM.docx]

**Model development**

We conducted RSF model with randomForestSRC package, Enet, Lasso, and Ridge with glmnet package, stepwise Cox model with survival package., CoxBoost model with CoxBoost package, plsRcox model with plsRcox package., SuperPC model with superpc package, GBM model with superpc package, and survival-SVM model with survivalsvm package. The optimized parameters were built from ntree and mtry with the best C-index value. The α of Enet was set to 0-1 (interval =0.1). Lasso is a regularization technique for linear regression which has attracted much attention in machine learning and statistics. The stepwise cox search direction was set to "both", "backward", and "forward". The penalty of Coxboost was identified by optimCoxBoostPenalty function. Stepwise regression can be used to optimize feature selection. For plsRcox, the size of components requested was calculated using the cv.plsRcox function. For supervised principal components, the superpc.cv method estimated a suitable feature threshold. Principal components method was used to reduce the dimensions. The index for number trees with the lowest cross-validation error was chosen by the cv.gbm function to bulid GBM model.
