# Supplementary figures and images for: Comprehensive machine learning-based preoperative blood features predict the prognosis for ovarian cancer
Source: BMC Cancer. 2024 Feb 26;24:267. doi: 10.1186/s12885-024-11989-1 (PMC10895771; doi:10.1186/s12885-024-11989-1)

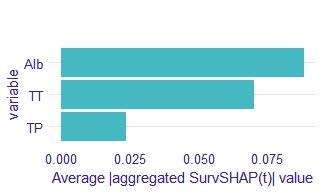


Figure S1 SHAP values of the optimal model.

Supplement: Supplementary file 2 — Supplementary Material 2 [file 12885_2024_11989_MOESM2_ESM.docx]
